# Patterns of seasonal and pandemic influenza-associated health care and mortality in Ontario, Canada

**DOI:** 10.1186/s12889-019-7369-x

**Published:** 2019-09-06

**Authors:** Michael Li, Benjamin M. Bolker, Jonathan Dushoff, Junling Ma, David J.D. Earn

**Affiliations:** 1 Department of Biology, McMaster University, Hamilton, ON L8S 4K1 Canada; 2 Department of Mathematics and Statistics, McMaster University, Hamilton, ON L8S 4K1 Canada; 3 M. G. deGroote Institute for Infectious Disease Research, McMaster University, Hamilton, ON L8S 4K1 Canada; 4 Department of Mathematics and Statistics, University of Victoria, Victoria, BC V8P 5C2 Canada

**Keywords:** Influenza, Pneumonia, Respiratory infections, Hospitalization, Mortality, Delay distributions

## Abstract

**Background:**

Mathematical and statistical models are used to project the future time course of infectious disease epidemics and the expected future burden on health care systems and economies. Influenza is a particularly important disease in this context because it causes annual epidemics and occasional pandemics. In order to forecast health care utilization during epidemics—and the effects of hospitalizations and deaths on the contact network and, in turn, on transmission dynamics—modellers must make assumptions about the lengths of time between infection, visiting a physician, being admitted to hospital, leaving hospital, and death. More reliable forecasts could be be made if the distributions of times between these types of events (“delay distributions”) were known.

**Methods:**

We estimated delay distributions in the province of Ontario, Canada, between 2006 and 2010. To do so, we used encrypted health insurance numbers to link 1.34 billion health care billing records to 4.27 million hospital inpatient stays. Because the four year period we studied included three typical influenza seasons and the 2009 influenza pandemic, we were able to compare the delay distributions in non-pandemic and pandemic settings. We also estimated conditional probabilities such as the probability of hospitalization within the year if diagnosed with influenza.

**Results:**

In non-pandemic [pandemic] years, delay distribution medians (inter-quartile ranges) were: Service to Admission 6.3 days (0.1–17.6 days) [2.4 days (-0.3–13.6 days)], Admission to Discharge 3 days (1.4–5.9 days) [2.6 days (1.2–5.1 days)], Admission to Death 5.3 days (2.1–11 days) [6 days (2.6–13.1 days)]. (Service date is defined as the date, within the year, of the first health care billing that included a diagnostic code for influenza-like-illness.) Among individuals diagnosed with either pneumonia or influenza in a given year, 19% [16%] were hospitalized within the year and 3% [2%] died in hospital. Among all individuals who were hospitalized, 10% [12%] were diagnosed with pneumonia or influenza during the year and 5% [5%] died in hospital.

**Conclusion:**

Our empirical delay distributions and conditional probabilities should help facilitate more accurate forecasts in the future, including improved predictions of hospital bed demands during influenza outbreaks, and the expected effects of hospitalizations on epidemic dynamics.

**Electronic supplementary material:**

The online version of this article (10.1186/s12889-019-7369-x) contains supplementary material, which is available to authorized users.

## Background

Influenza is a major global health concern that accounts annually for tens of thousands of deaths in North America [1, 2] and approximately 400,000 deaths worldwide [3]. Much effort has been invested in mathematical modeling of influenza dynamics in order to design improved control strategies [4, 5] and to estimate their economic impacts [6]. A fundamental limitation of such studies is a lack of quantitative information concerning the relationships between influenza infection and health care system utilization. In particular, how should we expect changes in influenza transmission dynamics induced by control measures [5, 7, 8] to translate into (i) changes in influenza-associated visits to physicians’ offices or hospitals and (ii) health care system burdens from influenza-associated hospitalizations?

Here we reduce this knowledge gap by exploiting an unusual data set made available to us through a special agreement with Statistics Canada. These data include linked health records over a number of years, allowing us to estimate the distributions of times from influenza diagnosis to hospital admission, and hospital admission to discharge or death.

## Methods

Access to anonymized individual health records was obtained through a Memorandum of Understanding (MoU) between the Ontario Ministry of Health and Long-term Care (MOHLTC) and Statistics Canada. The MoU defined a joint project on enhancing access to Ontario health data by the research community. Administrative databases were made available by MOHLTC to Statistics Canada, who facilitated record linkage by providing an encrypted health insurance number (EHIN) in each record. Files were made available to us in the Statistics Canada Research Data Centre (RDC) at McMaster University.

### Data sources

Three MOHLTC administrative databases were available to us: OHIP Ontario Health Insurance Program claims, as recorded in Medical Services Files. This database records all fee-for-service billings for *physician services* in Ontario; for each procedure, there is a *service code* and a *diagnostic code*. DAD Discharge Abstract Database, which contains hospital *inpatient records* (all data years) and *day procedure records* (data years prior to 2003/04), including diagnosis codes (one of which is flagged as “most responsible”). Inpatient records include the date of *admission* and the date of *discharge* or *death*. NACRS National Ambulatory Care Reporting System. Since 2003/04, Ontario hospitals have reported *day procedure events* to NACRS rather than the DAD.

All OHIP records included an EHIN, but a small number (≈2*%*) of DAD records were excluded from our analysis because an EHIN was not available (likely because the individuals in question were not Ontario residents at the time of hospitalization). For hospital events, we restricted attention to inpatients, so the NACRS database was not used.

### Data years

Access was provided to data from 1994 to 2010. However, format changes and the difficulty of working with large volumes of data using the computing infrastructure available in the RDC made it impractical to analyze all the data. The data years used in the present study were 2006/2007, 2007/2008, 2008/2009, and 2009/2010. Thus, the data covered the three influenza seasons preceding the 2009 pandemic and the pandemic year itself. The data file format was identical for these four years.

We defined the start and end dates of data years via the “influenza year”, running from 1 April to 30 March of the next year. Thus, for example, “data year 2006” refers to the time period from 1 April 2006 to 30 March 2007. This definition captures the typical seasonal influenza epidemic in the northern hemisphere from November to March.

### Relevant diagnosis codes

We restricted attention to records with diagnosis codes corresponding to influenza, pneumonia or other diseases of the respiratory system (Table 1). Ontario began to implement ICD10 (the tenth revision of the International Classification of Diseases) before our study period; however, the diagnosis codes recorded in the administrative databases used ICD9 for the duration of the study period. All records for individuals with at least one record with a code in Table 1 were included.

**Table 1:** ICD-9respiratorydiseasecodes for diagnoses included in the present study

Code	Definition
460–466	*Acute respiratory infections (ARI)*
460	Acute nasopharyngitis (common cold)
461	Acute sinusitis
462	Pharyngitis, acute
463	Tonsillitis, acute
464	Acute laryngitis and tracheitis
465	Acute upper respiratory infections of multiple or unspecified sites
466	Acute bronchitis and bronchiolitis
480–488	*Pneumonia and influenza (P&I)*
480	Viral pneumonia
486	Pneumonia, organism unspecified
487	Influenza
488	Influenza due to identified Avian influenza virus
488.1	Influenza due to identified 2009 H1N1 virus

Codes 481–485 were excluded because they indicate types of pneumonia not associated with influenza. Subcodes are not listed, except 488.1, which is highlighted because pandemic H1N1 is not normally considered a subset of “Avian influenza” and would not be expected to be found in this category

### Linked data

Linking the data by EHIN made it possible to obtain the anonymized individual-level temporal sequences of OHIP claims, hospital admissions, hospital discharges, and death, for Ontario residents during the data years investigated. First we filtered individuals with at least one influenza-like illness diagnosis in the OHIP database and aggregated to the earliest date within an influenza year (individuals with multiple claims in the full study period may be counted multiple times, but will only be counted once per influenza year). Then, we filtered all individuals with influenza-like illnesses that had a hospitalization record in DAD. We were therefore able to establish relationships between physician visits associated with influenza-like illness and hospitalization (even if influenza or pneumonia was listed in only one record of the sequence for a given patient). Note that individuals who have influenza-like illnesses and present with pneumonia are often given a diagnosis of pneumonia rather than influenza.

### Time distributions

In principle, it was straightforward to construct the desired distributions, i.e.,

(i) the distribution of times from physician diagnosis to hospitalization;
(ii) the distribution of times from hospitalization to discharge or death.

The primary practical challenge that we faced was working with the large event-data files.

### Time series

In order to contribute to understanding of seasonal patterns of respiratory illnesses, we aggregated events according to diagnosis and billing date. Because multiple billings are frequently associated with the same individual during a single illness, a given individual is likely to contribute to counts in multiple categories on multiple days during any given illness. Thus, the aggregated counts provide a time series of health service utilization (which counts the frequency of each diagnosis observed per day) rather than an epidemic time series (which would count each case of a disease once).

### Software

We used SAS to filter, link and extract data from the MOHLTC administrative databases available to us, and R (version 3.5.3) for all analyses. R packages glmmTMB (version 0.2.3, [9]) and depmixS4 (version 1.3-5, [10]) were used to fit Gamma and finite mixture Poisson distributions to the delay distributions (see Additional file 1).

## Results

### Data characteristics

Over the four data years (2006–2010), there were a total of 1.34 billion OHIP billing records. Of these, 31.9 million (2.39%) contained at least one of the ARI or P&I diagnosis codes listed in Table 1; after restricting attention to this subset, there were 7.60 million unique individuals in the database. During the same period, 4.27 million hospital inpatient stays were recorded in the DAD.

Most individuals were associated with a small number of billing records. Table 2 provides a quantile summary of the number of billing records (with diagnosis codes listed in Table 1) per person. Given the size of the data set (7.6 million individuals), the upper 2.5% tail in Table 2 (individuals with >17 billing records in the data set) corresponds to approximately 190,000 individuals.

**Table 2:** Quantile summary of the number of OHIP billing records per person for records with a diagnosis code in Table 1

2.5%	5%	25%	50%	75%	95%	97.5%	mean
1	1	1	3	5	13	17	4.201

Table 3 lists precise counts of records and patients, broken down by diagnoses of pneumonia or influenza (not for all diagnoses in Table 1) and by year.

**Table 3:** Detailed event and patient counts

	Code	Category	2006	2007	2008	2009
1	OHIPall	OHIP billing records	314,533,393	318,206,249	334,075,651	370,249,708
2	OHIPpi	∙P&I only	1,087,450	1,002,877	1,021,636	1,738,838
3	OHIPi	∙Influenza only	305,092	281,213	290,552	654,836
4	DADall	DAD/IP hospital records	1,068,648	1,068,888	1,065,756	1,070,806
5	upi	Unique EHINs with P&I in at least one OHIP record	467,746	440,606	445,195	627,733
6	ui	∙⊂upi; influenza	236,433	222,971	229,131	414,415
7	upiDAD	∙⊂upi; EHIN appears in a DAD/IP record	88,159	83,901	85,159	98,840
8	uiDAD	∙⊂upiDAD; influenza	20,042	18,738	18,894	33,039
9	DADu	Unique EHINs in DAD/IP records	826,314	828,860	827,458	831,011
10	OHIPupiDAD	OHIP records associated with unique EHINs in upiDAD	9,932,713	9,791,074	10,289,693	13,246,852
11	OHIPupiDADpi	∙⊂OHIPupiDAD; P&I	508,937	476,228	494,670	845,637
12	OHIPuiDAD	OHIP records associated with the unique EHINs in uiDAD	28,840	25,472	26,274	65,172
13	DADupiDAD	DAD/IP records associated with the unique EHIN list in upiDAD	114,146	137,229	139,459	157,247
14	DADuiDAD	DAD/IP records associated with the unique EHINs in uiDAD	27,849	25,886	25,819	44,514
15	DADd	deaths in DAD/IP records	40,396	40,567	41,761	41,142
16	DADdpi	∙P&I deaths within the year	11,241	11,239	11,715	11,516
17	DADdpi1	∙P&I deaths during first admission	7,117	7,190	7,495	7,396
18	DADdi	∙Influenza deaths within the year	600	587	642	928
19	DADdi1	∙Influenza deaths during first admission	401	402	422	620

Abbreviations: P&I = “pneumonia or influenza” (i.e., ICD-9 codes 486 or 487; see Table 1), OHIP = “Ontario Health Insurance Plan”, DAD/IP = “Discharge Abstract Database inpatient”, EHIN = “Encrypted Health Insurance Number”, ⊂ = “subset of”

From Table 3 it is straightforward to estimate various outcome probabilities given specific events having occurred during the focal data year (Table 4). For example, *P*(Hospitalized | P&I diagnosis) is defined as the probability that an individual who was diagnosed with pneumonia or influenza during a given influenza year was hospitalized during that year; the reason for hospitalization may not have been related to pneumonia or influenza. Similarly, *P*(P&I diagnosis | Hospitalized) denotes the probability that someone who was hospitalized during the influenza year was diagnosed with pneumonia or influenza that year; the P&I diagnosis might have occurred before, during or after the hospitalization. The one exception is the final row of Table 4, where *P*(Died in hospital | Hospitalized) denotes the probability that someone who was hospitalized during a given influenza year died *in hospital* that year, which could have happened only after hospitalization (though potentially after multiple distinct hospitalizations); note that *P*(Hospitalized | Died in hospital) would not be exactly 1 because some individuals who died in hospital in a given influenza year were hospitalized before the beginning of that influenza year.

**Table 4:** Estimated conditional probabilities of various outcomes

	Probability	2006	2007	2008	2009	Formula
1	*P*(Hospitalized | P&I diagnosis)	0.188	0.190	0.191	0.157	upiDAD/upi
2	*P*(Died in hospital | P&I diagnosis)	0.024	0.026	0.026	0.018	DADdpi/upi
3	*P*(Hospitalized | Influenza Diagnosis)	0.085	0.084	0.082	0.080	uiDAD/ui
4	*P*(Died in hospital | Influenza diagnosis)	0.003	0.003	0.003	0.002	DADdi/ui
5	*P*(P&I diagnosis | Hospitalized)	0.107	0.101	0.103	0.119	upiDAD/DADu
6	*P*(Died in hospital | Hospitalized)	0.049	0.049	0.050	0.050	DADd/DADu

The formulae for the probabilities are given in terms of the codes defined in Table 3

### Service billing by day of week

**Fig. 1:**
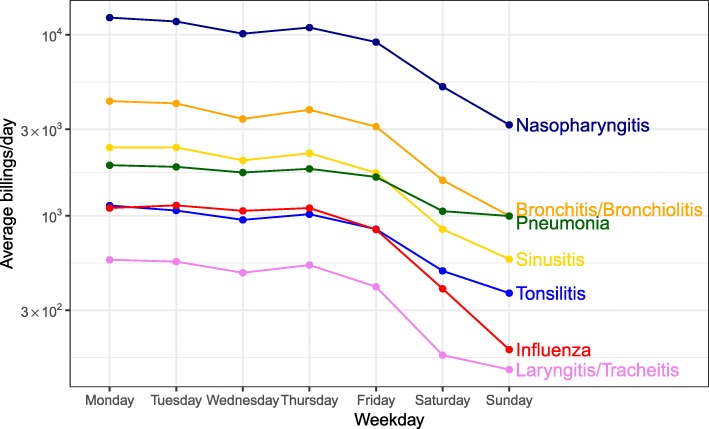
Service billings by day of week

The daily time series of billings show a small-amplitude weekly oscillation. Figure 1 shows that most acute respiratory illnesses have a characteristic weekly pattern of service billings (generally decreasing from Monday through Sunday, with a slight dip on Wednesday and a peak on Thursday). Pneumonia stands out as having smaller fluctuations over the course of the week.

### Seasonal patterns by week

**Fig. 2:**
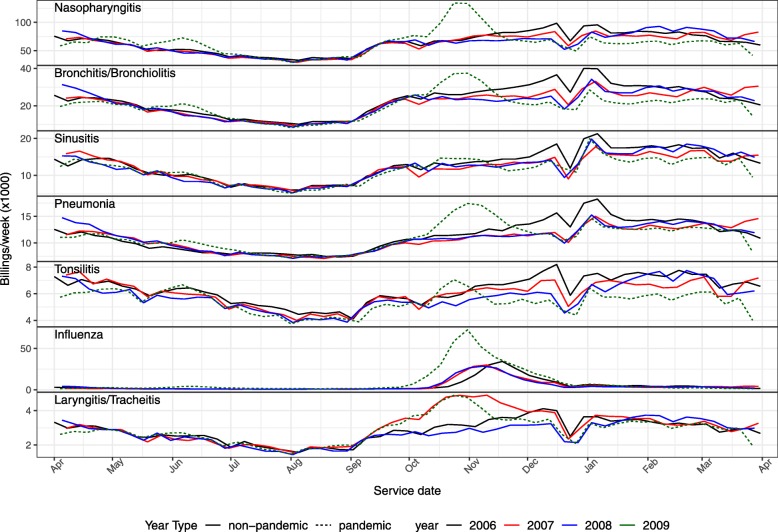
Weekly time series of service billings categorized by disease diagnosis

Figure 2 shows the weekly billings time series over each of the four influenza years in our data set. All the diagnoses display an annual oscillation. Pneumonia and tonsilitis have the least pronounced cycle while influenza has the most pronounced cycle. All diagnoses, including pneumonia, show a stronger seasonal peak during the 2009–2010 influenza season, which was dominated by pandemic H1N1. The dips just before the New Year are presumably driven by the holiday season (and associated delays in processing records), as is the case for well-known childhood disease time series [11].

We note that virological testing practices for influenza changed in Ontario during the 2009 pandemic. While fairly systematic testing was conducted for the first few months of the outbreak, testing restrictions were imposed in mid June 2009 [12]. The extent to which this policy change influenced the number of OHIP billing diagnoses is not clear.

### Time delay distributions

**Fig. 3:**
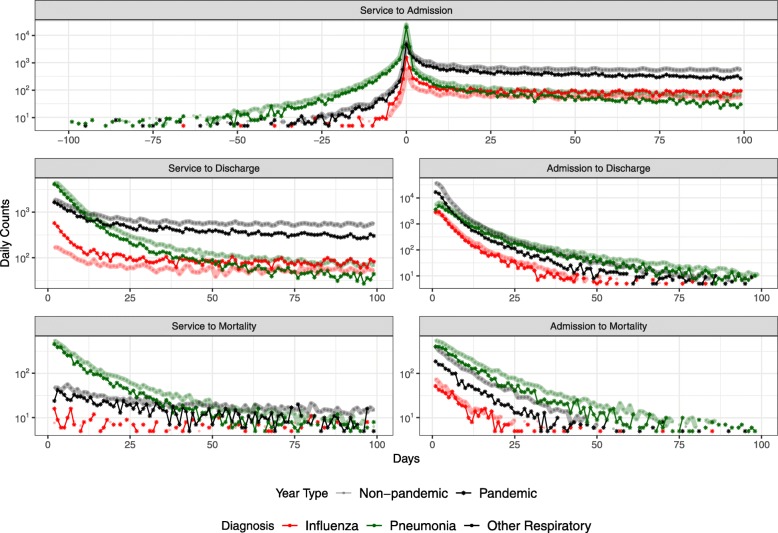
Time delay frequency distributions
- hospital admission to hospital discharge,
- hospital admission to death,
- service to hospital discharge,
- service to death.

Finally, we exploited the linked data to measure the elapsed time between various health-based outcomes. The resulting frequency distributions of these delays are shown in Fig. 3. We refer to the time of the first relevant diagnosis (influenza, pneumonia, or “other respiratory”) as the “service time”. The five panels of Fig. 3 show the frequency distributions of time delays for:

- service to hospital admission,

Figure 3 reveals that short delays (e.g., <30 days) are most common overall; the exceptions are a very long tail in the distribution of delays from service to admission, and a roughly flat distribution for influenza-related service to mortality (for which sample sizes may be too small to draw confident conclusions). Delay distributions are similar for influenza and “other respiratory” diseases; pneumonia generally has a longer time from admission to discharge or mortality. The latter pattern may be driven by the more substantial *negative* tail of delays from service to admission, which represents people who were admitted to hospital during the focal season but were first billed for services relating to one of the diagnostic codes listed in Table 1 some time *after* their admission.

**Fig. 4:**
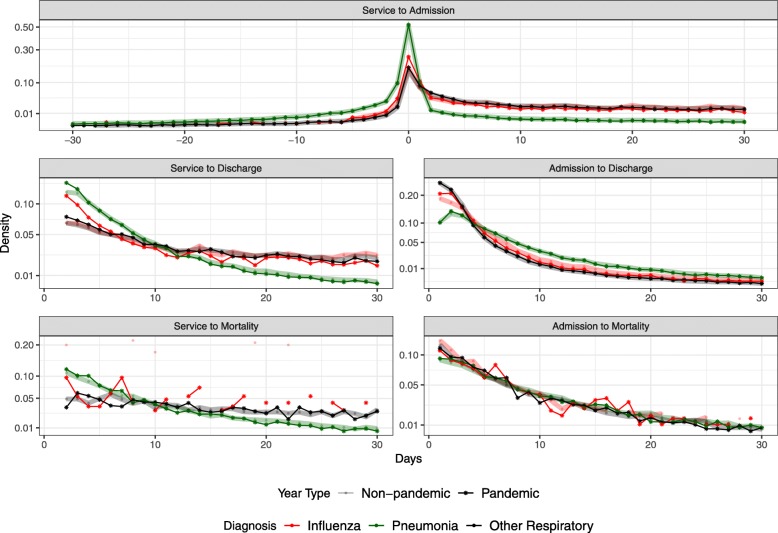
Time delay probability densities, obtained by normalizing the data shown in Fig. 3, restricted to events separated by at most 30 days

Normalizing the distributions in Fig. 3 by the total counts in the diagnostic category yields the estimated probability densities in Fig. 4; these densities are normalized, and shown, on a 30 day timescale rather than the 100 day timescale presented in Fig. 3 (in order to focus on pairs of events that are more likely to be causally connected). The leveling-off of the influenza distribution at a higher value than the pneumonia and other respiratory diagnoses in most of the plots (admission to discharge, admission to mortality) is an artifact of the smaller number of influenza cases; if the total number of cases is 10,000, then the non-zero values of the distribution cannot go below 10^−4^.

**Fig. 5:**
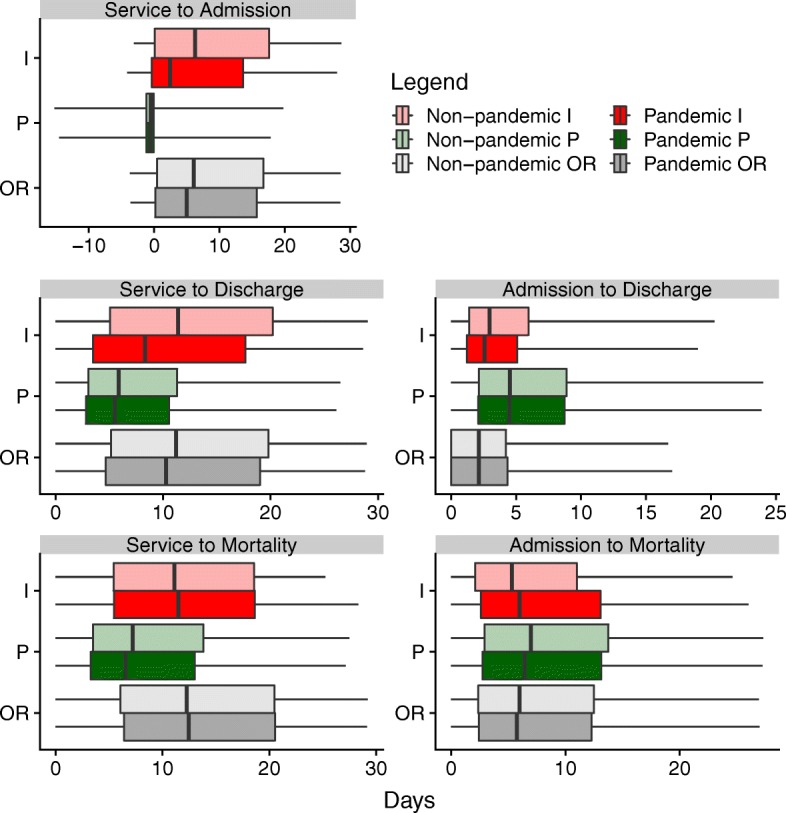
Quantiles of the delay distributions shown in Fig. 4

Figure 5 summarizes and facilitates comparison of the various delay distributions. Delays corresponding to the 2.5%, 25%, 50%, 75%, 97.5% quantiles are interpolated from the cumulative delay distributions.

In addition to summarizing the delay distributions by their quantiles, we also fitted parametric distributional models (see Additional file 1). Gamma models, using only two parameters (shape and scale), did a poor job fitting the delay distributions, which tend to have both sharp peaks and heavy tails relative to a Gamma distribution. We also fitted four-component finite mixtures of Poisson distributions; with 7 parameters (four rate parameters and three parameters determining the relative weights of the mixture components) these captured much more of the overall pattern, but still left some features unexplained. Other models, such as a finite mixture of negative binomial distributions, may provide better summaries; how complex a summary is most useful (e.g. a simple Gamma vs. a many-parameter finite mixture) will depend on particular applications. Additional file 1 provides the full details of the delay distributions fits. Full delay data are avalible at https://zenodo.org/record/3340201 for researchers wishing to derive other summaries of the data.

## Discussion

Infectious disease epidemics have substantial impacts on health care systems and economies. Mathematical models are extremely important for the purposes of planning [13], because the best we can do is to make decisions based on comparing the effects of various interventions on simulated epidemics (real experiments are usually impossible or unethical). For example, planners may need to decide whether to close schools [8, 14, 15] (and for how long to close them) or how to prioritize the use of limited supplies of vaccine [5].

In order predict the future time course of an epidemic, and the associated burden on societal structures, mathematical and statistical models require prior information [16, 17]. The most basic ingredients of such models are the mode of transmission and natural history of infection (latent, incubation and infectious periods). If we wish to predict patterns of health care system utilization, then we also need to quantify the relationship between the time of initial infection and the time at which health care resources (especially hospital beds) are likely to be exploited.

The time of initial infection is generally unobservable. However, for each influenza season that we examined, we were able to identify the time at which an individual was first *diagnosed* with influenza, pneumonia or another acute respiratory infection (Table 1). By taking advantage of database linkage, we were able to estimate several “delay distributions”, i.e., the distributions of time from initial diagnosis to hospital admission, discharge and death. Fortuitously, the data years available to us included the 2009 influenza pandemic, so we were also able to compare distributions in non-pandemic years with a pandemic year.

Furthermore, we were able to compare various outcome probabilities given specific events (P&I diagnosis, hospitalization, and death) during different years. We emphasize that these conditional probabilities reflect associations, not causal links. Neverthless, they are useful for identifying patterns in the data that may be valuable to explore more thoroughly in the future. For example, we found that the probability of hospitalization for an individual who was diagnosed with pneumonia or influenza during the pandemic year was, surprisingly, lower than in non-pandemic years; mortality in hospital was roughly the same for both pandemic and non-pandemic years.

Regardless of whether the style of modelling is detailed and individual-based with quantitative goals [18] or abstract with qualitative goals [19], the delay distributions that we have estimated—and made available electronically in online additional material—provide a useful empirical backbone.

### Limitations

Our study was restricted to the Canadian province of Ontario, a region where the population is primarily concentrated in a large megalopolis (the Greater Toronto Area). The extent to which the delay distributions we have estimated can be applied to regions other than Ontario is not known. However, it is reasonable to expect that they are similar in other parts of Canada, and in many other industrialized countries. Ideally, it would be better to repeat our study using data for particular populations of interest.

## Conclusion

Forecasting of influenza epidemics and associated patterns of health care system utilization can be conducted with greater confidence using the empirically estimated delay distributions that we have presented here (Figs. 3, 4 and 5; data in Additional file 1). While quantifying delay distributions was our primary goal, we were able to obtain a number of statistics that are of independent interest (Table 4). For example, approximately 5% of people admitted to hospital died there, and if they died in hospital then the probability that they had been diagnosed with influenza or pneumonia within the year was 10%.

The primary challenge in conducting the kind of research presented here is the size of the data sets and the requirement to analyze them in a secure environment. As computing power and the sophistication and availability of software for analyzing “big data” increases, much more extensive studies of linked individual-level health-related databases should become feasible.

## Additional file

Additional file 1 Additional file 1 provides the full details of the parametric fits of the delay distributions. (PDF 134 kb)

## Data Availability

All summary data and parametric fitting code used in our analyses are publicly available at https://zenodo.org/record/3340201.

## Abbreviations

ARI: Acute respiratory infections
DAD: Discharge Abstract Database
EHIN: Encrypted Health Insurance Number
MOHLTC: Ontario Ministry of Health and Long-Term Care
NACRS: National Ambulatory Care Reporting System
OHIP: Ontario Health Insurance Program
P&I: Pneumonia and influenza
RDC: Statistics Canada Research Data Centre
