# Supplementary material for: Patterns of seasonal and pandemic influenza-associated health care and mortality in Ontario, Canada
Source: BMC Public Health. 2019 Sep 6;19:1237. doi: 10.1186/s12889-019-7369-x (PMC6731609; doi:10.1186/s12889-019-7369-x)
Supplement: Supplementary file 1 — Additional file 1 provides the full details of the parametric fits of the delay distributions. (PDF 134 kb) [file 12889_2019_7369_MOESM1_ESM.pdf]

Delay density fitting

In the main text, we presented the empirical delay distributions. Here, we present parametric fits to the delay data using two approaches: a Gamma distribution using a generalized linear model approach, and a (four-component) finite mixture of Poisson distributions using maximum likelihood estimation. Table A1 lists all Gamma and mixture Poisson parameter estimates of each type.

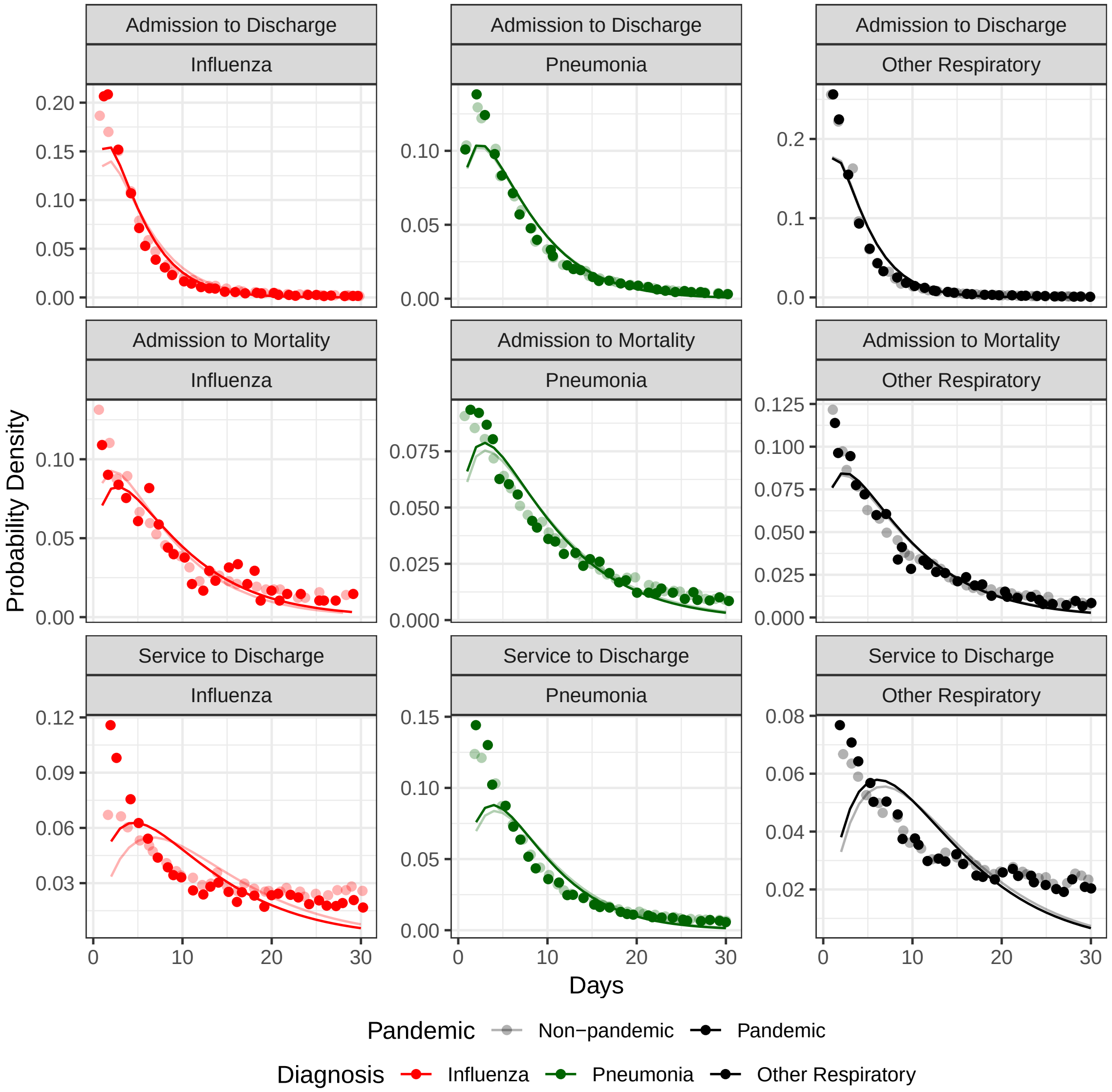

Figure A1: Gamma delay fits.

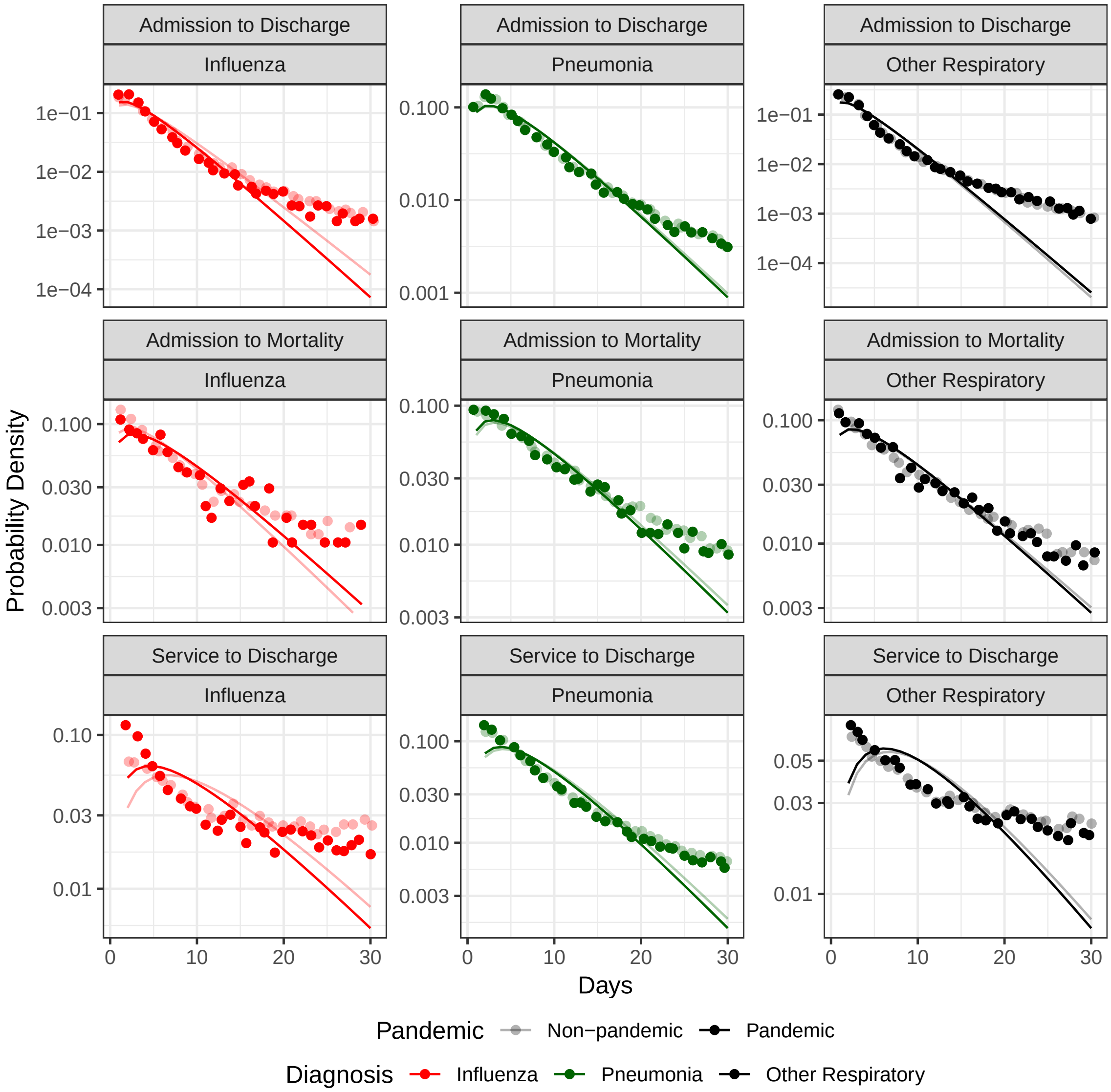

Figure A2: Gamma delay fits (log scale)

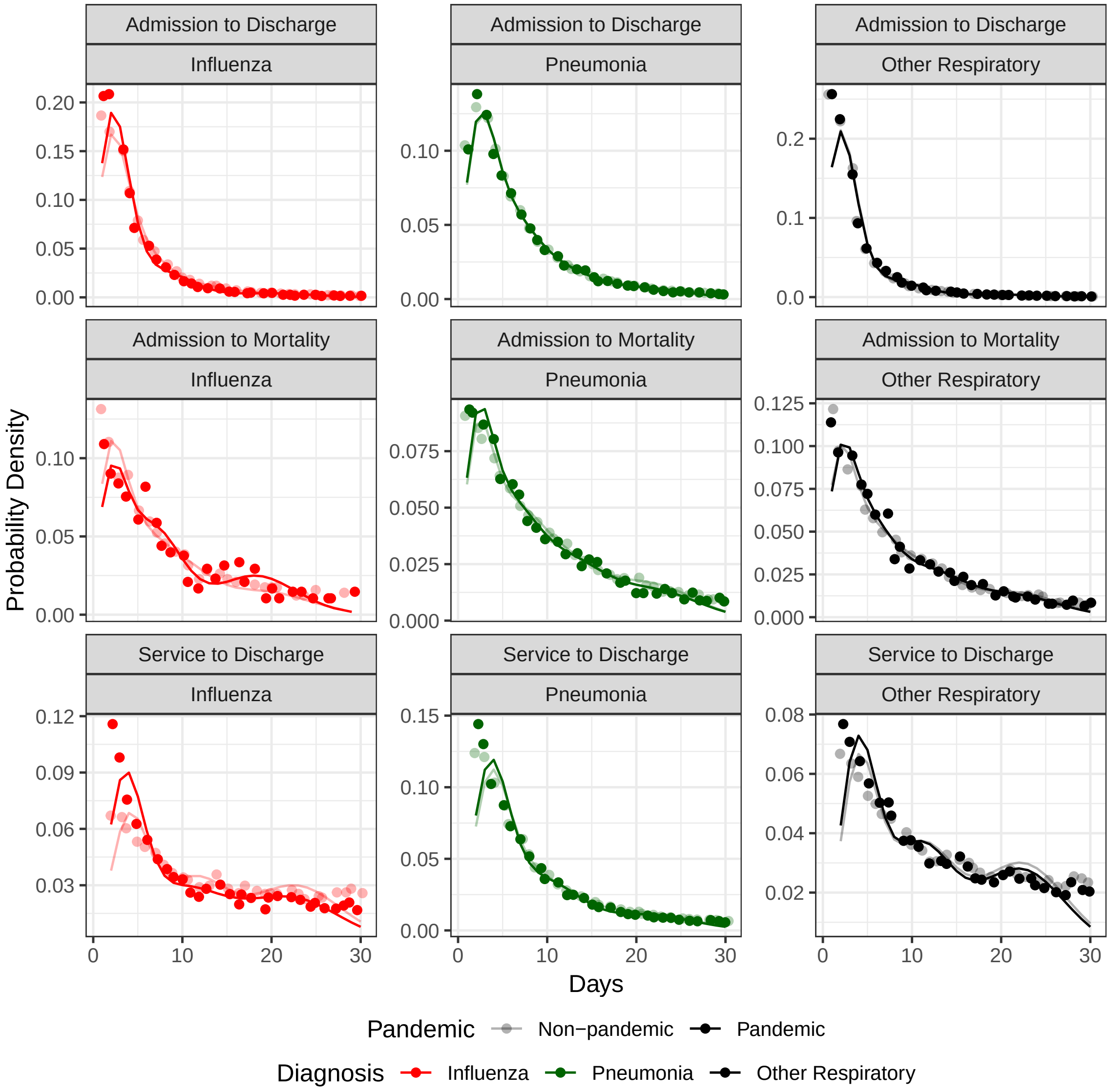

Figure A3: Mixture-Poisson delay fits.

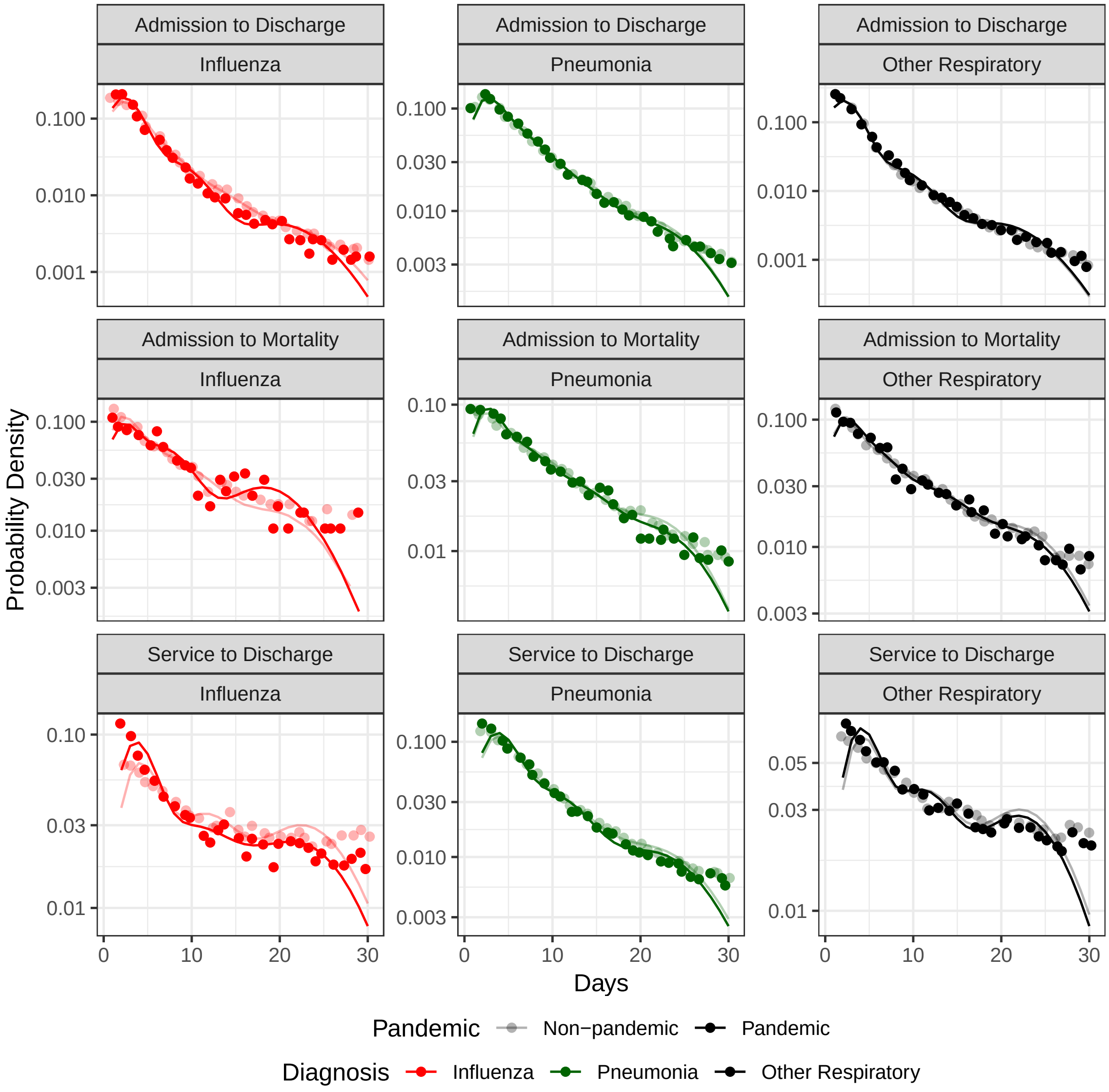

Figure A4: Mixture-Poisson delay fits (log scale)

Table A1: Estimated parameters for parametric delay fits. G_shape and G_scale are the shape and scale parameters for Gamma fits, and MP 1-4 are mean and relative weights parameter (in parentheses) for each component.

Pandemic	Diagnosis	Type	G_shape	G_scale	MP 1	MP 2	MP 3	MP 4
Non-pandemic	Influenza	Admission to Discharge	1.46	3.54	0.97 (0.64)	1.87 (0.23)	2.54 (0.09)	3.09 (0.04)
Non-pandemic	Influenza	Admission to Mortality	1.38	5.76	0.92 (0.40)	1.81 (0.23)	2.39 (0.22)	3.01 (0.15)
Non-pandemic	Influenza	Service to Discharge	1.98	6.77	1.53 (0.36)	2.50 (0.29)	3.15 (0.17)	3.15 (0.18)
Non-pandemic	Other Respiratory	Admission to Discharge	1.49	2.70	0.94 (0.52)	0.94 (0.31)	2.19 (0.13)	3.00 (0.03)
Non-pandemic	Other Respiratory	Admission to Mortality	1.34	6.61	0.90 (0.36)	1.86 (0.26)	2.51 (0.22)	3.10 (0.16)
Non-pandemic	Other Respiratory	Service to Discharge	2.03	6.54	1.52 (0.35)	2.47 (0.30)	3.13 (0.13)	3.13 (0.22)
Non-pandemic	Pneumonia	Admission to Discharge	1.53	4.61	1.08 (0.49)	1.93 (0.28)	2.51 (0.15)	3.07 (0.09)
Non-pandemic	Pneumonia	Admission to Mortality	1.46	6.60	1.02 (0.34)	1.99 (0.28)	2.57 (0.21)	3.11 (0.18)
Non-pandemic	Pneumonia	Service to Discharge	1.89	4.60	1.44 (0.55)	2.22 (0.20)	2.61 (0.13)	3.11 (0.13)
Pandemic	Influenza	Admission to Discharge	1.48	3.13	1.01 (0.42)	1.01 (0.36)	2.16 (0.18)	3.02 (0.05)
Pandemic	Influenza	Admission to Mortality	1.44	6.14	0.98 (0.36)	1.94 (0.18)	2.12 (0.19)	2.93 (0.27)
Pandemic	Influenza	Service to Discharge	1.66	6.91	1.41 (0.45)	2.34 (0.18)	2.73 (0.12)	3.15 (0.26)
Pandemic	Other Respiratory	Admission to Discharge	1.47	2.78	0.93 (0.47)	0.93 (0.34)	2.17 (0.14)	3.00 (0.04)
Pandemic	Other Respiratory	Admission to Mortality	1.38	6.29	0.95 (0.36)	1.86 (0.28)	2.57 (0.21)	3.11 (0.14)
Pandemic	Other Respiratory	Service to Discharge	1.94	6.51	1.50 (0.37)	2.46 (0.30)	3.12 (0.13)	3.13 (0.19)
Pandemic	Pneumonia	Admission to Discharge	1.54	4.51	1.08 (0.50)	1.95 (0.29)	2.55 (0.14)	3.09 (0.08)
Pandemic	Pneumonia	Admission to Mortality	1.44	6.41	1.03 (0.36)	1.97 (0.28)	2.61 (0.21)	3.13 (0.15)
Pandemic	Pneumonia	Service to Discharge	1.86	4.45	1.42 (0.59)	2.25 (0.14)	2.50 (0.15)	3.10 (0.12)
